# Laboratory High-Contrast X-ray Microscopy of Copper Nanostructures Enabled by a Liquid-Metal-Jet X-ray Source

**DOI:** 10.3390/nano14050448

**Published:** 2024-02-29

**Authors:** Kristina Kutukova, Bartlomiej Lechowski, Joerg Grenzer, Peter Krueger, André Clausner, Ehrenfried Zschech

**Affiliations:** 1 deepXscan GmbH, Zeppelinstr. 1, 01324 Dresden, Germany; 2 Fraunhofer Institute for Ceramic Technologies and Systems, Maria-Reiche-Str. 5, 01099 Dresden, Germany; 3 Research Area Nanomaterials, Brandenburg University of Technology Cottbus-Senftenberg, Konrad-Zuse-Str. 1, 03046 Cottbus, Germany

**Keywords:** X-ray microscopy, radiography, image contrast, nanostructure, physical failure analysis, copper interconnects

## Abstract

High-resolution imaging of Cu/low-k on-chip interconnect stacks in advanced microelectronic products is demonstrated using full-field transmission X-ray microscopy (TXM). The comparison of two lens-based laboratory X-ray microscopes that are operated at two different photon energies, 8.0 keV and 9.2 keV, shows a contrast enhancement for imaging of copper nanostructures embedded in insulating organosilicate glass of a factor of 5 if 9.2 keV photons are used. Photons with this energy (Ga-Kα radiation) are generated from a Ga-containing target of a laboratory X-ray source applying the liquid-metal-jet technology. The 5 times higher contrast compared to the use of Cu-Kα radiation (8.0 keV photon energy) from a rotating anode X-ray source is caused by the fact that the energy of the Ga-Kα emission line is slightly higher than that of the Cu-K absorption edge (9.0 keV photon energy). The use of Ga-Kα radiation is of particular advantage for imaging of copper interconnects with dimensions from several 100 nm down to several 10 nm in a Cu/SiO_2_ or Cu/low-k backend-of-line stack. Physical failure analysis and reliability engineering in the semiconductor industry will benefit from high-contrast X-ray images of sub-μm copper structures in microchips.

## 1. Introduction

With the growing complexity and on-chip interconnect density of leading-edge microelectronic devices, backend-of-line (BEoL) physical failure analysis (PFA) in the semiconductor industry is facing new challenges and requirements. High-resolution imaging of metal interconnect structures in BEoL stacks, with dimensions from several 100 nm down to several 10 nm, is needed for process development, failure analysis, quality control and reliability engineering. To identify irregularities in the dimensions of metal interconnects and to detect defects are requests to PFA labs in the semiconductor industry. Today’s PFA workflows for imaging of on-chip copper interconnects, embedded in SiO_2_ or organosilicate glass (OSG), a so-called low-k material, usually include destructive cross-sectioning of the BEoL stack by applying focused ion beam (FIB) milling and subsequent imaging using scanning electron microscopy (SEM), as well as in some cases transmission electron microscopy (TEM) [1]. With this FIB-SEM/TEM workflow, buried structures and defects are reliably imaged, however, at the cost of destroying the sample.

Transmission X-ray microscopy (TXM) has been proven to be a compelling imaging technique applied for materials characterization, for the study of materials aging and materials degradation kinetics, for the 3D study of biological cells, and for many other applications. Nano-scale structures have been imaged at synchrotron radiation beamlines and in laboratories [2,3]. Full-field X-ray microscopy is characterized by a larger field of view compared to transmission electron microscopy (TEM). One essential advantage of the use of X-rays instead of electrons for microscopic imaging is the larger penetration depth of X-rays, i.e., micrometers to millimeters, depending on the photon energies used. Consequently, the sub-μm on-chip interconnects do not need to be cut, i.e., the copper structures remain embedded in the insulating dielectrics, and the interfaces—including the barrier layers—are not affected. This advantage has been demonstrated for the imaging of BEoL structures [4], as well as for studying kinetic processes such as electromigration [5] and crack propagation [6]. X-ray imaging with high spatial resolution, particularly with lens-based full-field X-ray microscopes, is a unique technique for nondestructive imaging of sub-μm structures. High-resolution X-ray imaging of BEoL structures of microchips has been demonstrated [6]. However, the image contrast is relatively weak for usually used photon energies, i.e., 8.0 keV (Cu-Kα radiation) and 5.4 keV (Cr-Kα radiation). Contrast enhancement applying phase contrast for imaging BEoL structures was demonstrated, e.g., at a synchrotron radiation beamline for 4 keV photon energy [7].

Another way to increase the contrast for X-ray imaging significantly is choosing a photon energy slightly above the X-ray absorption edge of a chemical element that is a constituent of a component in the object to be imaged. The choice of the photon energy is straight forward, using synchrotron radiation because of the tunability of the photon energy within a large energy range. For laboratory X-ray microscopes that employ characteristic radiation for imaging (not the polychromatic bremsstrahlung), an anode target material has to be selected for the X-ray source, that has a characteristic X-ray line slightly above the X-ray absorption edge of the chemical element of interest. For imaging of 3D copper nanostructures, e.g., 3D copper interconnects embedded in SiO_2_ or organosilicate glass, the use of Ga-Kα photons with an energy of 9.2 keV is beneficial since this characteristic X-ray line is about 0.2 keV above the Cu-K absorption edge at a photon energy of 9.0 keV. Despite this significant relevance for microelectronics, this photon energy has been rarely exploited because Ga is not an anode target material used in solid anode or rotating anode X-ray sources for laboratory X-ray microscopes. Recently, the first laboratory TXM experiments using Ga-Kα radiation (9.2 keV photon energy) from a Ga or Ga-In target of a liquid-metal-jet X-ray source have been reported. Fella et al. built up a laboratory X-ray microscope and discussed the potential of TXM using a liquid-metal-jet X-ray source with a Ga target [8,9], and Sutherland et al. demonstrated the imaging of sub-μm copper interconnects of microchips with a modified commercial X-ray microscope (Ultra, Carl Zeiss, Pleasanton, CA, USA) customized with a liquid-metal-jet X-ray source (D2+, Excillum, Kista, Sweden) operating at photon energies of 9.2 keV [10]. Very recently, the first results from the laboratory X-ray microscope used in this study were published, using In-Kα radiation (24.2 keV photon energy) from a Ga-In alloy target of a liquid-metal-jet X-ray source [11]. A laboratory nano-XCT tool combining the electron beam of a scanning electron microscope (SEM) with the precise, broadband X-ray detection of a superconducting transition-edge sensor (TES) microcalorimeter, was described in [12]. Pt-Lα photons with an energy of 9.4 keV, i.e., also slightly above the Cu-K absorption edge, were used to image Cu/SiO_2_ structures in an integrated circuit [12].

**Figure 1:**
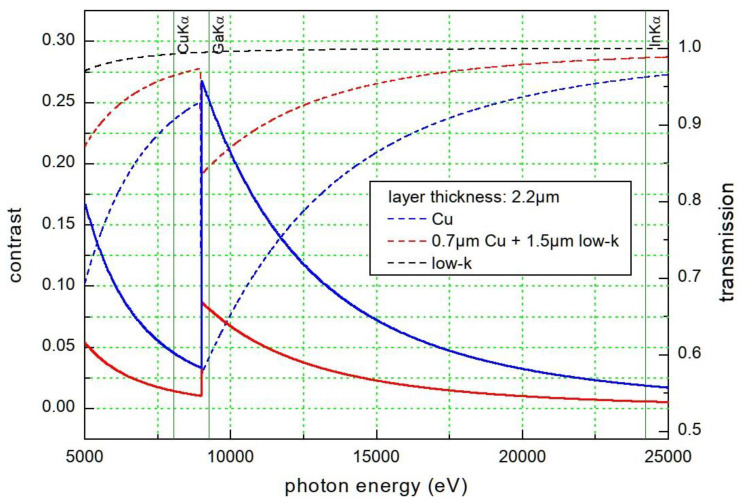
Contrast (solid lines) and X-ray transmission (dotted lines) through 2.2 µm material: pure components (Cu—blue lines, low-k material—black lines) and composite—red line. The contrast is the ratio of the differences of the transmissions/calculated intensities divided by their sum: contrast 1 means that one material is opaque, contrast 0 that both materials have equal transmission.

To motivate our studies presented in this paper, the change of the X-ray transmission (right y axis) as a function of photon energy (x axis) through a 2.2 µm thick composite stack consisting of about 30 vol.-% Cu and 70 vol.-% OSG, and for the extreme values in beam direction of pure Cu or pure OSG, is shown in Figure 1. The following densities were used for the calculation of the X-ray transmission as a function of photon energy: 8.921 g·cm^−3^ for the patterned electroplated copper thin films [13], and 0.92 g/cm^3^ for the low-k material with the composition Si 37.4 wt.-%, C 18.3 wt.-%, O 44.3 wt.-% (density of the dense dielectrics 1.32 g·cm^−3^ [14]) and a porosity of 30%. (see below). The CXRO database was used for the calculation of the X-ray transmission of these materials [15].

It is clearly seen that the image contrast (left y axis) between the components of the Cu/low-k composite is enhanced by a factor of about 5 for imaging with Ga-Kα radiation compared to Cu-Kα radiation, because of the sudden drop of the X-ray transmission at the Cu-K absorption edge at 9.0 keV, which is located between these two X-ray emission lines. Since liquid-metal-jet X-ray sources usually have Ga or Ga-In targets, these X-ray sources exploiting Ga-Kα radiation are beneficial for high-contrast imaging of copper nanostructures. In addition, the anode concept for compact X-ray sources based on the liquid-metal-jet technology [16] allows us to apply a higher power density to the anode, resulting in an X-ray flux and a brightness that are higher than those of conventional microfocus X-ray sources [17].

One of the main challenges for state-of-the-art laboratory TXM is to guarantee that the photon flux remains high enough to obtain a good signal-to-noise ratio of the image. To mitigate these limitations, i.e., long data acquisition times and low image contrast, photons with an energy of 9.2 keV generated from a Ga-In target of a liquid-metal-jet X-ray source were used for high-resolution imaging of 3D copper interconnect structures in this study. The advantage of X-ray microscopy using photons with an energy slightly above the Cu-K absorption edge for imaging of on-chip Cu interconnects of advanced microelectronic products is shown. The contrast enhancement is demonstrated by comparing similar structures imaged at 8.0 keV and 9.2 keV photon energy, respectively.

## 2. X-ray Microscopy with Ga-Kα Radiation

**Figure 2:**
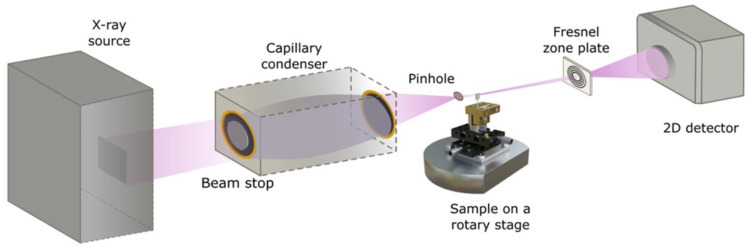
Scheme of the laboratory transmission X-ray microscope run at 9.2 keV photon energy.

A laboratory full-field X-ray microscope [18] (dXs S50, deepXscan, Dresden, Germany) equipped with a liquid-metal-jet X-ray source (D2+, Excillum, Kista, Sweden) with an indium-rich Ga-In target, a glass capillary as condenser optic (Sigray, Concord, CA, USA) that illuminates the sample, a magnifying Fresnel zone plate (FZP) as objective lens (Applied Nanotools, Edmonton, AB, Canada) and a scintillator-coupled CMOS camera as detector (Crycam™, Crytur, Turnov, Czech Republic) was used. The beam path in the transmission X-ray microscope is depicted in Figure 2. Furthermore, we placed a pinhole between the condenser and the sample to minimize stray light. Moreover, we evacuated most of the beam path to mitigate X-ray beam intensity reduction due to scattering and absorption. The X-ray source was run at an acceleration voltage for the electrons of 40 kV and an emission current of ~3.75 mA, resulting in a power output of about 150 W. The size of the electron beam at the anode was 80 × 20 μm^2^, resulting in a projection of the X-ray spot diameter of about 20 µm. It should be noted that the used In-rich alloy containing 47 wt.-% Ga, 37 wt.-% In and 16 wt.-% Sn is not optimal for the number of Ga-Kα photons generated; however, it was the alloy practically available for the experiments in this study. The use of a Ga-rich alloy with 95 wt.-% Ga and 5 wt.-% In as a target material yields a Ga-Kα radiant flux of 1.1 × 10^7^ photons/(s × mrad^2^) when operated at 70 kV and 20 µm nominal spot size [19]. It is about a factor of 3 more compared to the In-rich alloy used in this study—about 4.0 × 10^6^ photons/(s × mrad^2^). The ratio is expected to be similar at an acceleration voltage for the electrons of 40 kV.

To demonstrate the advantage of using Ga-Kα radiation to improve the image contrast for copper nanostructures that are isolated by dielectrics (low-k materials), images of the same object were taken for comparison using an X-ray microscope (nanoXCT-100, Xradia, Concord, CA, USA) with an X-ray source with rotating Cu anode target [4]. The X-ray source was run at an acceleration voltage for the electrons of 40 keV and an emission current of 30 mA at an electron focal spot of 700 × 70 µm^2^, resulting in a projected X-ray spot diameter of about 70 µm.

**Figure 3:**
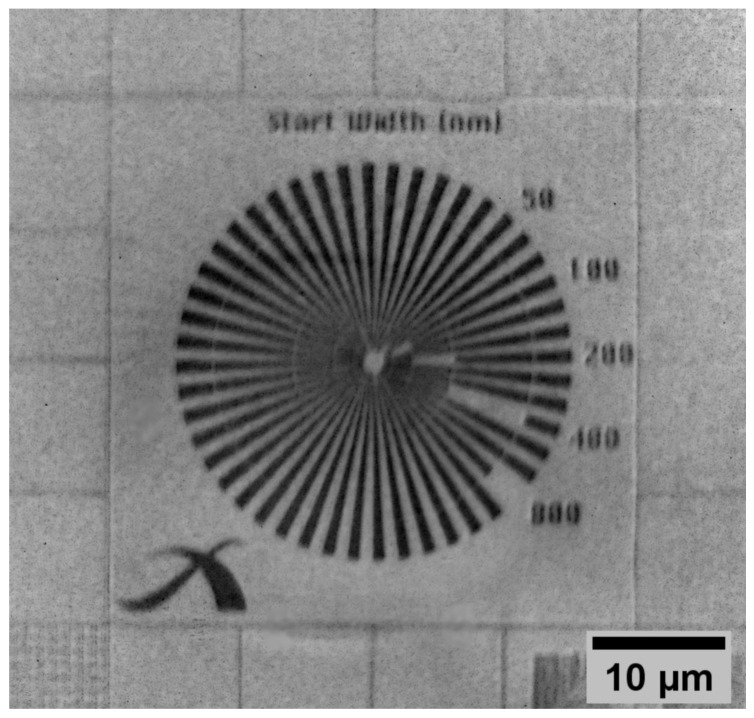
X-ray image of a Siemens star test structure (photon energy 9.2 keV, average of 10 images, acquisition time for each image 120 s, background and flatfield corrected, no denoising, 1024 px × 1024 px). The notches in the imaged Siemens star design start with a width of 50 nm (in the center) and are increased clockwise gradually to 100 nm, 200 nm, 400 nm and finally 800 nm.

The imaging performance of the used transmission X-ray microscope (dXs S50, deepXscan, Dresden, Germany) is demonstrated at the X-ray resolution target, possessing a Siemens star test structure with a minimal feature size of 50 nm, patterned into a 700 nm thick Au absorber. With the present setup, we achieved a total magnification of 200× (FZP 20×, camera objective 10×). Figure 3 shows the resulting image of the Siemens star pattern, based on 10 individual radiographs. The exposure time of each of the collected images was 120 s with camera pixel binning 2 (1024 px × 1024 px). The background-corrected stacked images were subsequently divided by an adequate flat field image collected with the same parameter settings. From the visual impression, 100 nm features are resolved in the radiograph.

## 3. Laboratory X-ray Microscopy—Imaging of Copper Interconnects

The transmission X-ray microscope run with a liquid-metal-jet X-ray source as described above was applied for imaging of on-chip copper interconnects embedded in organosilicate glass. To demonstrate the capabilities of the TXM with this advanced liquid metal Ga-In X-ray source, a cross-section sample from a microchip manufactured in 14 nm CMOS technology node was prepared using focused ion beam (FIB) milling, resulting in a lamellae with a thickness of 2.2 µm. This sample includes an on-chip interconnect stack comprising 12 layers of copper with layer thicknesses from 80 nm (metal 1, M1) to 1.5 µm (metal 12, M12). The insulating low-k dielectrics is an organosilicate glass that was processed using chemical vapor deposition (CVD) and subsequent UV curing [20].

**Figure 4:**
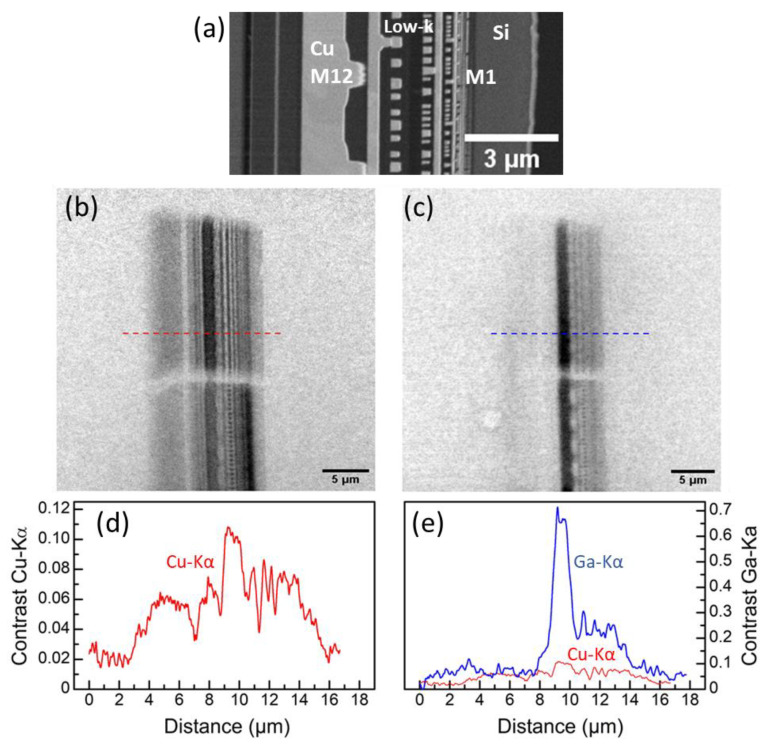
(**a**) SEM image of a FIB cross-section through the Cu/low-k stack of a microchip, manufactured in 14 nm CMOS technology node, (**b**,**c**) radiographs for the identical stack using Cu-Kα radiation (**b**) and Ga-Kα radiation (**c**,**d**,**e**) corresponding line plots of the contrast across the layer stack (marked positions as dashed lines), red for Cu-Kα (**d**) and for comparison (**e**), blue for Ga-Kα (**e**).

Figure 4 shows a SEM image of the Cu/low-k stack of the microchip as described above (a), as well as radiographs imaged using Cu-Kα radiation and Ga-Kα radiation, respectively, (b) and (c). In addition, corresponding line plots (d) and (e) of the image contrast (calculation see above) taken at the marked dashed lines in (b) and (c) are provided. Both radiographs represent an average of 3 images (1024 px × 1024 px), each acquired with 120 s exposure time. The dashed lines indicate the selected position for the line plots of the image contrast along the Cu/low-k stack, using 16-bit images. Figure 4d,e provides a comparison of the image contrast using Cu-Kα and Ga-Kα radiation, indicated by red and blue color. Figure 4e shows clearly that the image contrast in the radiographs of the Cu/low-k stack with copper interconnect structures is significantly improved if Ga-Kα radiation is used instead of Cu-Kα radiation (the Cu-Kα contrast line (red) is shown additionally to the Ga-Ka contrast line (blue), for comparison). For Cu metal 12 (M12), it is about 5 times higher using Ga-Kα radiation compared to Cu-Kα radiation. This experimental result confirms the theoretical calculation, visualized by the contrast leap for copper at the Cu-K absorption edge (see Figure 1).

## 4. Conclusions

Full-field X-ray microscopy with Ga-Kα radiation enables the imaging of copper nanostructures in engineered materials and systems with high image contrast. Choosing a liquid-metal-jet X-ray source with a Ga-containing target is mitigating the limitations of state-of-the-art laboratory X-ray microscopes, i.e., long data acquisition times caused by low photon flux and low image contrast. This advantage was demonstrated, i.e., high-contrast imaging of on-chip copper interconnects embedded in organosilicate glass (low-k material) was shown. Compared with state-of-the-art laboratory TXM applying Cu-Kα radiation, imaging copper nanostructures in on-chip interconnect stacks applying Ga-Kα radiation provides an image contrast that is improved by about a factor of 5, with the consequence that fewer photons are needed for high-quality imaging. In addition, the use of the liquid-metal-jet technology potentially enables the application of a higher power density to the anode, resulting in a higher X-ray flux. In summary, the shorter exposure time for the imaging of copper nanostructures is of high relevance for the semiconductor industry.

The use of Ga-Kα radiation is not limited to samples from microelectronic products. X-ray microscopy at 9.2 keV photon energy can be advantageously adapted to the study of other materials systems with Cu-containing components as well.

## Data Availability

Data are contained within the article.
